# Association of COVID-19 Infection and SARS-CoV-2 Vaccination With Echocardiographic Pericardial Involvement in a Real-World Outpatient Cohort

**DOI:** 10.7759/cureus.113147

**Published:** 2026-07-22

**Authors:** Fabrizio Salvucci, Lea Petrella, Beatrice Foroni, Gabriella Grassi, Maria Luisa Anti, Nicolita Nitisoara, Adriana Coppola, Livio Luzi, Roberto Codella, Carmine Gazzaruso, Giuseppe Di Fede

**Affiliations:** 1 Internal Medicine, Istituto di Medicina Biologica, Milan, ITA; 2 Department of Methods and Models for Economics, Territory, and Finance (MEMOTEF), Sapienza University of Rome, Rome, ITA; 3 Internal Medicine, Istituto Clinico Beato Matteo del Gruppo San Donato, Vigevano, ITA; 4 Department of Biomedical Sciences for Health, University of Milan, Milan, ITA; 5 Department of Psychoneuroendocrine Immunology, Università Europea di Roma (EUR), Milan, ITA

**Keywords:** covid-19, echocardiography, inflammatory myopericardial syndrome, myopericarditis, pericarditis, sars-cov-2 vaccination

## Abstract

Background

During the COVID‑19 pandemic, inflammatory myopericardial syndromes have been reported following SARS‑CoV‑2 infection and SARS‑CoV‑2 vaccination. However, the prevalence of such conditions in routine clinical practice, including mild or residual pericardial alterations, remains incompletely characterized. This study aimed to evaluate the association between prior SARS-CoV-2 infection, SARS-CoV-2 vaccination, and echocardiographic pericardial involvement in a real-world outpatient cohort undergoing routine transthoracic echocardiography.

Methods

This retrospective cohort study analyzed 1,431 consecutive patients undergoing routine outpatient echocardiography from 2018 to 2022 at the Istituto di Medicina Biologica di Milano, Milan, Italy. Pericardial involvement was defined as echocardiographic findings consistent with pericardial inflammation, including pericardial effusion, thickening, hyperechogenicity, and fibrinous strands, encompassing not only clinically evident pericarditis but also minimal pericardial alterations and residual findings. Multivariable logistic regression and average marginal effects were used to assess associations with prior COVID‑19 infection, SARS‑CoV‑2 vaccination status, age, sex, and BMI, adjusting for calendar year and confounders.

Results

Pre‑pandemic pericardial involvement was detected in 101/459 (22%) examinations (2018-2019), rising to 463/673 (68.8%) during the pandemic years (2020-2022; P < 0.001). Both prior COVID‑19 infection (odds ratio (OR) 1.99, 95% confidence interval (CI) 1.17-3.43, P = 0.012) and vaccination (OR 2.08, 95% CI 1.29-3.35, P = 0.003) were independently associated with pericardial involvement, with a significant interaction suggesting that prior infection attenuated additional risk from vaccination, and vice versa. Female sex, older age, and higher BMI were also associated with higher odds.

Conclusions

In this real-world echocardiography cohort, both SARS-CoV-2 infection and vaccination were associated with increased odds of echocardiographic pericardial involvement, including minimal and residual alterations. The marked temporal rise during the pandemic may reflect a combination of subclinical inflammatory changes and evolving referral and diagnostic practices, while the absolute risk of vaccine-associated myopericarditis remained low compared with the overall benefits of vaccination.

## Introduction

COVID-19 has been associated with an increased risk of long-term cardiovascular diseases, including cerebrovascular events, arrhythmias, ischemic heart disease, heart failure, thromboembolic disease, and myopericarditis [1-3]. Population-based studies report up to a two-fold higher risk of cardiovascular events among survivors compared to pre-pandemic viral illnesses, even in non-hospitalized individuals, suggesting persistent subclinical myocardial or endothelial injury [2-4]. Among these complications, myopericarditis is likely underdiagnosed and may evolve into a chronic condition with relevant medium- and long-term consequences [3,5]. SARS-CoV-2 infection has been associated with a marked increase in myocarditis and pericarditis incidence, with many cases remaining asymptomatic and detectable only through advanced imaging, such as cardiac magnetic resonance [4,6]. Myopericarditis has also been reported following SARS-CoV-2 vaccination, particularly with mRNA-based vaccines [4,7-8]. Although post-vaccination myocarditis is rare, typically affecting young males and showing a favorable prognosis compared with infection-related forms, its underlying mechanisms, potentially involving immune activation pathways, remain under investigation [4,7-8].

The 2025 European Society of Cardiology (ESC) Guidelines introduced the concept of “inflammatory myopericardial syndrome” (IMPS), reflecting the shared pathophysiology and frequent overlap between myocarditis and pericarditis [5]. Both post-infectious and post-vaccination forms may be more prevalent than recognized due to mild or subclinical presentations and limitations of passive surveillance systems [4]. Importantly, pericardial involvement ranges from clinically overt pericarditis to minimal or residual pericardial alterations detectable only by imaging (small effusions, mild thickening, and increased echogenicity). Such minimal findings may reflect prior inflammatory injury and are often not captured by symptom‑driven surveillance. Echocardiography is a widely available and cost-effective tool for detecting pericardial abnormalities.

This study aimed to assess the association between prior COVID-19 infection, SARS-CoV-2 vaccination status, and echocardiographic pericardial abnormalities in patients undergoing routine echocardiographic evaluation across pre-pandemic and pandemic periods.

## Materials and methods

Study design

This retrospective cohort study analyzed consecutive patients who underwent transthoracic echocardiography at the Istituto di Medicina Biologica di Milano, an outpatient cardiology clinic Milan, Italy, between January 2018 and December 2022. The study period was chosen to include both pre‑pandemic years (2018-2019) and pandemic years (2020- 2022), allowing temporal comparison of the prevalence of echocardiographic pericardial abnormalities.

Population and sample

The study population consisted of consecutive adults aged 18 years or older who underwent transthoracic echocardiography at our outpatient cardiology clinic between January 2018 and December 2022. Patients were not included as part of a universal screening program; rather, echocardiography was performed according to standard clinical indications, including routine cardiovascular assessment, follow-up of known cardiac conditions, and evaluation of new symptoms. The final analytic sample included 1,431 patients after exclusion of individuals with incomplete data, poor-quality echocardiographic images, or known pre-existing pericardial disease. In our sample, no missing data were in the dataset. All patients aged 18 years or older who underwent echocardiography during the study period were eligible. Exclusion criteria were incomplete demographic or clinical data, echocardiographic images of insufficient quality for diagnostic assessment, or known pre‑existing pericardial disease documented before 2018.

Data collection

Clinical and demographic data were abstracted retrospectively from electronic medical records. Demographic variables included age (years), sex (male/female), and body mass index (BMI, kg/m²), calculated from height and weight recorded at the time of echocardiography. Clinical history variables included documented COVID‑19 infection status (confirmed by PCR or antigen test) and SARS‑CoV‑2 vaccination status (number of doses received: 0, 1, 2, or ≥3). COVID‑19 infection status was defined as a documented positive PCR or rapid antigen test before echocardiography. Vaccination status was obtained from regional vaccination registries and patient‑reported information, with verification by official documentation when available. For dose‑specific analyses, doses were categorized as 0 (unvaccinated), 1, 2, or ≥3. The specific vaccine type (mRNA‑based or other) was not consistently recorded and was therefore not included in the analysis.

Echocardiographic assessment and echocardiographic pericardial abnormalities

All examinations were performed using standard transthoracic echocardiography equipment by experienced sonographers, in accordance with established guidelines. Images were acquired in standard parasternal long‑axis, parasternal short‑axis, apical four‑chamber, apical two‑chamber, and subcostal views. Pericardial assessment included pericardial thickness, echogenicity, presence and quantification of pericardial effusion, and signs of hemodynamic compromise. Echocardiographic pericardial abnormalities were diagnosed based on echocardiographic findings consistent with pericardial inflammation, including (1) new or increased pericardial effusion compared with prior studies, (2) pericardial thickening >3 mm, (3) increased or hyperechogenic pericardial echotexture, and (4) fibrinous strands or adhesions within the pericardial space. The diagnosis encompassed acute pericarditis (symptoms <4 weeks), subacute pericarditis (four to six weeks), chronic pericarditis (>3 months), and pericarditis in remission with residual echocardiographic findings. For this study, we operationally defined "pericardial involvement" or “echocardiographic pericardial abnormalities” to include both clinically manifest pericarditis and minimal or residual pericardial alterations (small pericardial effusion, pericardial thickening ≤ or slightly >3 mm, hyperechogenicity, or fibrinous strands) even in the absence of typical clinical symptoms or ECG/biomarker changes. These echo‑only findings were recorded as possible evidence of prior or ongoing pericardial inflammation and included in the binary outcome. All studies were reviewed by board‑certified cardiologists experienced in echocardiography. In cases of equivocal findings, a consensus reading was performed by two independent reviewers. Pericardial involvement presence/absence was recorded as a binary variable for each patient.

Statistical analysis

Descriptive statistics were calculated for all variables. Continuous data are presented as medians with interquartile ranges (IQRs) due to non‑normal distributions; categorical data are reported as frequencies and percentages. Pre‑pandemic (2018-2019) and pandemic (2020-2022) periods were compared using a two‑sample test for equality of proportions for pericardial involvement prevalence. Pearson’s chi‑square test was used for univariate associations between categorical variables and pericardial involvement. The Mann‑Whitney U test was used to compare continuous variables between patients with and without pericardial involvement. Multivariable logistic regression models were used to evaluate how individual characteristics and clinical history influenced the likelihood of pericardial involvement. The primary model included age (continuous), sex, BMI (continuous), COVID‑19 infection status, vaccination status (vaccinated vs. unvaccinated), year of examination (2018 as reference), and an interaction term between COVID‑19 infection and vaccination status to assess whether the effect of vaccination on echocardiographic pericardial abnormalities risk differed by infection history. A secondary model replaced binary vaccination status with the number of vaccine doses (0, 1, 2, ≥3), adjusting for the same covariates and including interaction terms between COVID‑19 infection and each dose category to evaluate dose‑specific effects stratified by infection status. Both models yielded adjusted odds ratios (ORs) with 95% confidence intervals (CIs); statistical significance was defined as a two‑tailed P < 0.05. Model fit was assessed using the Hosmer‑Lemeshow goodness‑of‑fit test and residual plots, and multicollinearity was evaluated using variance inflation factors (VIF < 5 considered acceptable). To improve interpretability, average marginal effects were computed following Muller and MacLehose. Adjusted predicted probabilities of pericarditis were estimated for different combinations of COVID-19 infection and vaccination status, averaged over the distribution of other covariates. Average marginal effects were expressed as adjusted predicted probabilities for each combination of exposure status, and pairwise contrasts between strata were reported as ORs with 95% CIs. For the dose-specific model, marginal effects compared each dose level with lower dose categories within infection strata. Sensitivity analyses assessed robustness of findings by (1) restricting analyses to patients without prior cardiovascular disease, (2) excluding patients with BMI extremes (<18.5 or >40 kg/m²), and (3) analyzing pre‑pandemic and pandemic periods separately. All analyses were conducted in R (version 4.5.0) using the packages stats, emmeans, and ggplot2.

Ethical considerations

The study was conducted in accordance with the Declaration of Helsinki. Retrospective analysis of de-identified clinical data was approved by the Istituto di Medicina Biologica, Ethics Committee for Scientific Research (September 3, 2025; Protocol no. 03). Informed consent was waived because of the retrospective design and the use of anonymized/de-identified data. Access to the data was restricted to authorized investigators, and confidentiality protections complied with applicable institutional and national data protection standards.

## Results

Study population characteristics

Of the 1,431 patients included, 679/1,431 (47.4%) received a pericardial abnormality diagnosis based on echocardiographic findings. The overall cohort had a median age of 60 years (IQR 47-72) and a median BMI of 24.9 kg/m² (IQR 22.0-28.4); 876/1,431 (61.2%) were male and 555/1,431 (38.8%) were female. Males were slightly older than females (median 60 vs. 59 years) and had a higher median BMI (25.5 vs. 24.2 kg/m²). However, the prevalence of echocardiographic pericardial abnormality differed markedly by sex, with females showing a substantially higher proportion than males (405/749, 54.1% vs. 274/682, 40.2%; P < 0.001), consistent with previously reported sex differences in pericardial disease presentation. Of the 1,431 patients included, 883 (61.7%) had no history of COVID-19 infection and were unvaccinated, 285 (19.9%) were vaccinated but had no prior infection, 155 (10.8%) had a prior COVID-19 infection and were unvaccinated, and 108 (7.5%) had both a prior infection and had received at least one vaccine dose.

Temporal trends in pericarditis prevalence

In the pre‑pandemic period (2018-2019), the proportion of echocardiographic pericardial involvement diagnoses remained relatively stable at 101/459 (22.0%). A marked increase was observed during the pandemic years (2020-2022), with 463/673 (68.8%) patients receiving an echocardiographic pericardial abnormality diagnosis (P < 0.001 compared with the pre‑pandemic period). This temporal shift prompted further multivariable analysis to assess the contribution of demographic and clinical factors. Note that reported prevalences reflect this inclusive definition and therefore encompass both clinically overt pericarditis and isolated minimal pericardial alterations detected at echocardiography. To further explore these associations, univariate analyses were performed, as shown in Table 1. Table 1 summarizes the univariate associations between pericardial involvement and the main clinical and demographic variables. Pearson’s chi-square test showed significant associations for COVID-19 infection, vaccination status, sex, age group, and BMI group, supporting their inclusion in the multivariable models.

**Table 1:** Univariate associations between pericarditis and clinical variables Pearson’s chi-square test was used for categorical variables.

Variable	Pearson χ²	df	P-value
COVID-19 infection	62.22	1	<0.001
Vaccination status	137.95	1	<0.001
Sex	27.09	1	<0.001
Age group	62.02	2	<0.001
BMI group	16.47	2	<0.001

Multivariable logistic regression analysis

In the primary multivariable logistic regression model (Table 2), patients with a previous COVID‑19 infection had a significantly higher probability of echocardiographic pericardial abnormalities compared with those who had never been infected (OR 1.99, 95% CI 1.17-3.43; P = 0.012).

**Table 2:** Adjusted odds ratios with 95% confidence intervals from the primary multivariable logistic regression model predicting pericarditis

Variable	OR	95% CI lower	95% CI upper	P-value
Intercept	0.007	0.003	0.017	<0.001
COVID-19 infection	1.993	1.166	3.425	0.012
Vaccination	2.078	1.290	3.349	0.003
Age (per year)	1.038	1.030	1.046	<0.001
Male sex	0.568	0.441	0.729	<0.001
BMI (per unit)	1.067	1.041	1.094	<0.001
Year 2019	0.723	0.452	1.150	0.172
Year 2020	1.855	1.250	2.774	0.002
Year 2021	6.953	4.215	11.606	<0.001
Year 2022	6.728	3.633	12.632	<0.001
COVID × vaccination	0.485	0.230	1.028	0.058

Vaccination status was also significantly associated with echocardiographic pericardial abnormalities, with vaccinated individuals showing higher odds (OR 2.08, 95% CI 1.29-3.35; P = 0.003). The interaction term between COVID‑19 infection and vaccination status showed a trend toward significance but did not reach the 0.05 threshold (OR 0.485, 95% CI 0.230-1.028; P = 0.058). Age was systematically associated with increased risk: each additional year corresponded to a 3.8% increase in odds (OR 1.038, 95% CI 1.030-1.046; P < 0.001). BMI was also positively associated with the outcome, with a 6.7% higher odds per one‑unit increase (OR 1.067, 95% CI 1.041-1.094; P < 0.001). By contrast, male gender was associated with lower odds compared with females (OR 0.568, 95% CI 0.441-0.729; P < 0.001). The effect of calendar year revealed a marked increase beginning in 2020. Relative to 2018 (reference), the odds of pericardial involvement were nearly twice as high in 2020 (OR 1.855, 95% CI 1.250-2.774; P = 0.002) and increased sharply in 2021 (OR 6.953, 95% CI 4.215-11.606; P < 0.001) and 2022 (OR 6.728, 95% CI 3.633-12.632; P < 0.001). The year 2019 did not differ significantly from 2018 (OR 0.723, 95% CI 0.452-1.150; P = 0.172), supporting the association of increased echocardiographic pericardial abnormalities prevalence with the onset of the COVID‑19 pandemic rather than with the pre‑pandemic period alone.

Model diagnostics

**Figure 1:**
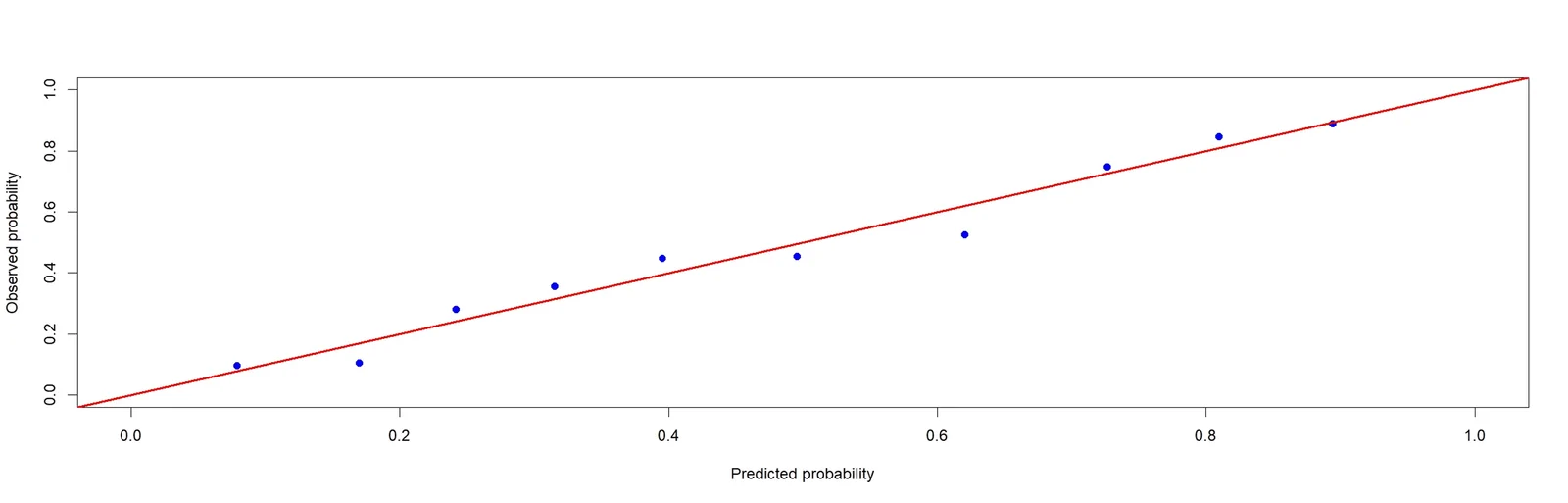
Calibration plot between the predicted and observed probabilities across the range of the predicted risk

Multicollinearity assessment via variance inflation factors (VIFs) indicated no problematic collinearity among model covariates. All GVIF values were below the pre-specified threshold of 5, with the highest value observed for calendar year (GVIF = 3.58), followed by vaccination status (GVIF = 2.89) and COVID-19 infection status (GVIF = 2.88), reflecting the expected temporal correlation between pandemic exposures and calendar year without compromising model stability. Model discrimination was formally assessed using the area under the ROC curve (AUC, or C-statistic), computed via the pROC package in R. The primary logistic regression model achieved an AUC of 0.809 (95% CI 0.787-0.831, DeLong method), indicating good discriminative performance. Model calibration was assessed using the Hosmer-Lemeshow goodness-of-fit test with 10 groups (χ² = 17.02, df = 8, P = 0.030). Although the test was statistically significant, visual inspection of the calibration plot showed generally good agreement between predicted and observed probabilities across the range of predicted risk, with only modest deviations in the intermediate-risk range, supporting acceptable calibration of the primary model (Figure 1).

Model discrimination was assessed using the area under the receiver operating characteristic curve (AUC), which was 0.809 (95% CI 0.787-0.831), indicating good discriminative ability.

Average marginal effect analysis

To facilitate interpretation of the primary model, we computed average marginal effects for the probability of pericardial involvement across all combinations of vaccination status (vaccinated vs. unvaccinated) within COVID‑19 infection status (infected vs. not infected) (Table 3).

**Table 3:** Average marginal effects for the vaccination status according to the COVID-19 infection status

Contrast	COVID-19 status	Adj. prob. unvaccinated	Adj. prob. vaccinated	Odds ratio	SE	P-value
Vaccinated vs. unvaccinated	Negative	35.7% (95% CI 31.3–40.3%)	53.5% (95% CI 44.0–62.8%)	2.078	0.117	0.003
Vaccinated vs. unvaccinated	Positive	52.5% (95% CI 41.4–63.4%)	52.7% (95% CI 39.6–65.4%)	1.008	0.299	0.979

Among individuals without prior COVID‑19 infection, vaccination was associated with higher odds of echocardiographic pericardial abnormalities (OR 2.078, 95% CI 1.17-3.43; P = 0.003). By contrast, no meaningful difference was observed between vaccinated and unvaccinated individuals with a history of COVID‑19 infection (OR 1.008, 95% CI 0.299-3.42; P = 0.979). The adjusted predicted probabilities of pericardial involvement, averaged over the distribution of covariates, are reported for each combination of COVID-19 and vaccination status. Among COVID-19-negative patients, the probability of pericardial involvement was 35.7% (95% CI 31.3-40.3%) in unvaccinated individuals and 53.5% (95% CI 44.0-62.8%) in vaccinated individuals. Among COVID-19-positive patients, adjusted probabilities were similar regardless of vaccination status: 52.5% (95% CI 41.4-63.4%) in unvaccinated and 52.7% (95% CI 39.6-65.4%) in vaccinated individuals. We then repeated the comparison reversing the exposure, assessing the odds of echocardiographic pericardial abnormalities between COVID‑positive and COVID‑negative subjects within vaccinated and unvaccinated groups (Table 4).

**Table 4:** Average marginal effects for COVID-19 infection status according to the vaccination status

Contrast	Vaccination status	Adj. prob. COVID-negative	Adj. prob. COVID-positive	Odds ratio	SE	P-value
COVID-positive vs. COVID-negative	Unvaccinated	35.7% (95% CI 31.3–40.3%)	52.5% (95% CI 41.4–63.4%)	1.993	0.138	0.012
COVID-positive vs. COVID-negative	Vaccinated	53.5% (95% CI 44.0–62.8%)	52.7% (95% CI 39.6–65.4%)	0.967	0.293	0.905

Among unvaccinated individuals, prior COVID‑19 infection was associated with a significant increase in the odds of echocardiographic pericardial abnormalities (OR 1.993, 95% CI 1.25-3.42; P = 0.012). By contrast, no meaningful difference was observed between COVID‑positive and COVID‑negative individuals within the vaccinated group (OR 0.967, 95% CI 0.29-3.39; P = 0.905). Considering COVID-19 infection status as the exposure of interest, among unvaccinated patients, the adjusted probability of pericardial involvement was 35.7% (95% CI 31.3-40.3%) in COVID-19-negative individuals and 52.5% (95% CI 41.4-63.4%) in COVID-19-positive individuals. Among vaccinated patients, probabilities were again similar between COVID-19-negative and COVID-19-positive individuals (53.5% (95% CI 44.0-62.8%) vs. 52.7% (95% CI 39.6-65.4%)), consistent with the non-significant contrast reported in Table 3.

Dose‑response relationship analysis

We then fitted a complementary logistic regression model examining the number of SARS‑CoV‑2 vaccine doses (0, 1, 2, or ≥3) instead of a binary vaccination indicator, adjusting for age, gender, BMI, year of observation, and COVID‑19 infection status (Table 5).

**Table 5:** Adjusted odds ratios with 95% confidence intervals from the dose-response model predicting pericarditis

Variable	OR	95% CI lower	95% CI upper	P-value
Intercept	0.007	0.003	0.017	<0.001
COVID-19 infection	1.971	1.152	3.392	0.014
1 dose	2.093	1.047	4.289	0.039
2 doses	2.145	1.258	3.686	0.005
≥3 doses	1.846	0.871	4.082	0.118
Age (per year)	1.038	1.030	1.046	<0.001
Male sex	0.568	0.441	0.729	<0.001
BMI (per unit)	1.067	1.041	1.094	<0.001
Year 2019	0.723	0.452	1.151	0.173
Year 2020	1.856	1.250	2.774	0.002
Year 2021	6.910	4.188	11.535	<0.001
Year 2022	6.958	3.691	13.316	<0.001
COVID × 1 dose	0.596	0.206	1.754	0.342
COVID × 2 doses	0.335	0.123	0.919	0.032
COVID × ≥3 doses	0.595	0.177	2.144	0.410

Compared with no vaccination, receiving one or two doses was associated with a significant increase in odds of echocardiographic pericardial abnormalities: one dose (OR 2.093, 95% CI 1.047-4.289; P = 0.039) and two doses (OR 2.145, 95% CI 1.258-3.686; P = 0.005). By contrast, three or more doses did not show a statistically significant association (OR 1.846, 95% CI 0.871-4.082; P = 0.118). Prior COVID‑19 infection remained a significant predictor (OR 1.971, 95% CI 1.152-3.392; P = 0.014). The model included interaction terms between COVID‑19 infection and each dose category. The interaction was not significant for one dose (P = 0.342) or for three or more doses (P = 0.410). However, for two doses, the interaction was significant (OR 0.335, 95% CI 0.123-0.919; P = 0.032), suggesting that the effect of two doses on pericarditis odds was attenuated in individuals with prior COVID‑19 infection. The demographic covariates behaved consistently with the primary model: age showed a modest but significant increase in echocardiographic pericardial abnormalities odds per year (OR 1.038, 95% CI 1.030-1.046; P < 0.001), male sex was associated with lower odds (OR 0.568, 95% CI 0.441-0.729; P < 0.001), and BMI remained positively associated (OR 1.067, 95% CI 1.041-1.094; P < 0.001). The year effect echoed the earlier model, with odds of pericardial abnormality rising markedly in 2020 (OR 1.856, 95% CI 1.250-2.774; P = 0.002) and dramatically in 2021 (OR 6.910, 95% CI 4.188-11.535; P < 0.001) and 2022 (OR 6.958, 95% CI 3.691-13.316; P < 0.001). Average marginal effects were computed for all combinations of COVID‑19 infection status (positive/negative) and number of doses (0, 1, 2, ≥3) (Table 6).

**Table 6:** Average marginal effects for the number of doses according to the COVID‑19 infection status

Contrast	COVID-19 status	Odds ratio	SE	P-value
1 dose vs. 0 doses	Negative	2.093	0.171	0.166
2 doses vs. 0 doses	Negative	2.145	0.128	0.027
≥3 doses vs. 0 doses	Negative	1.846	0.212	0.400
2 doses vs. 1 dose	Negative	1.025	0.351	1.000
≥3 doses vs. 1 dose	Negative	0.882	0.515	0.993
≥3 doses vs. 2 doses	Negative	0.861	0.456	0.981
1 dose vs. 0 doses	Positive	1.248	0.330	0.950
2 doses vs. 0 doses	Positive	0.718	0.603	0.871
≥3 doses vs. 0 doses	Positive	1.099	0.480	0.998
2 doses vs. 1 dose	Positive	0.576	0.929	0.730
≥3 doses vs. 1 dose	Positive	0.880	0.697	0.997
≥3 doses vs. 2 doses	Positive	1.529	0.400	0.900

Among COVID‑negative individuals, those who received two doses had substantially higher odds of echocardiographic pericardial abnormality compared with unvaccinated subjects (OR 2.145; P = 0.027). No other dose comparisons were statistically significant within the COVID‑negative group. By contrast, among individuals with prior COVID‑19 infection, none of the dose contrasts reached statistical significance, indicating that vaccination status did not meaningfully alter pericardial abnormality risk in this subgroup.

## Discussion

This five‑year retrospective cohort study of 1,431 consecutive patients undergoing echocardiography revealed a dramatic temporal increase in pericardial abnormality diagnoses coinciding with the COVID‑19 pandemic, rising from 101/459 (22.0%) in pre‑pandemic years to 463/673 (68.8%) during pandemic years. Both COVID‑19 infection and vaccination were independently associated with increased pericardial involvement risk, with approximately twofold increases in odds for each exposure. Importantly, the association between vaccination and echocardiographic pericardial abnormalities was primarily observed among individuals without prior COVID‑19 infection, while those with prior infection showed elevated pericardial involvement risk regardless of vaccination status. These findings provide important real‑world evidence regarding the cardiovascular impact of both SARS‑CoV‑2 infection and vaccination in routine clinical practice [8].

Temporal trends and pandemic impact

The most striking finding of this study is the marked increase in echocardiographic pericardial abnormality diagnoses during pandemic years, with odds ratios exceeding sixfold in 2021 and 2022 compared to 2018. This temporal pattern strongly suggests a causal relationship with the COVID‑19 pandemic, encompassing both direct effects of SARS‑CoV‑2 infection and potential vaccine‑related effects. Our findings are consistent with epidemiologic studies demonstrating that SARS‑CoV‑2 increased the incidence of myocarditis and pericarditis at least 15‑fold over pre‑COVID levels [1]. Several mechanisms may contribute to this dramatic increase. First, direct viral infection of cardiac tissue has been documented in COVID‑19 patients, with myocardial damage observed in approximately 80% of cases with elevated serum IL‑6 [9]. The characteristic infiltrate in COVID‑19 myopericarditis consists predominantly of CD68+ macrophages with fewer T cells, indicating a robust innate immune response [10]. Second, the systemic inflammatory response associated with COVID‑19, characterized by hyperactivation of complement and TLR4‑related pathways, may contribute to cardiac inflammation even in the absence of direct viral invasion [11,12]. Third, persistent arrhythmias and subclinical myocardial injury have been demonstrated in 40-60% of post‑COVID patients, suggesting ongoing inflammatory processes that may manifest as pericarditis during follow‑up echocardiography [13,14].

Studies using cardiac MRI have detected myocarditis in patients one to six months after acute SARS‑CoV‑2 infection, even in those with initially mild symptoms [6]. This delayed presentation may explain the sustained elevation in echocardiographic pericardial abnormalities throughout 2021 and 2022, reflecting both acute and post‑acute sequelae of infection. Our finding that COVID‑19 infection is associated with a two‑fold increase in the odds of echocardiographic pericardial abnormalities (OR 1.99) aligns with the growing evidence of long‑term cardiovascular complications following SARS‑CoV‑2 infection [1]. Xie et al. reported a significantly increased one‑year incidence of cardiovascular events among 153,760 COVID‑19 survivors compared with controls, including ischemic heart disease, heart failure, arrhythmias, and inflammatory heart disease, even in non‑hospitalized individuals [1]. Importantly, our data suggest that this elevated risk persists in the unvaccinated group but is attenuated among those subsequently vaccinated, consistent with observational evidence that complete vaccination is associated with a lower burden of post‑COVID cardiovascular events [15,16]. The mechanisms underlying post‑COVID cardiovascular complications are multifactorial. Direct endothelial damage from viral infection, persistent low‑grade inflammation, autoimmune phenomena, and microvascular thrombosis have been implicated in both acute and post‑acute cardiovascular sequelae [17]. Endothelial dysfunction and ongoing vascular inflammation may persist for months after apparent viral clearance, contributing to myocardial and pericardial involvement even in patients without overt cardiac symptoms. CD68+ macrophage infiltration, a hallmark of COVID‑19‑related myopericarditis, reflects sustained innate immune activation and is consistent with this model of chronic low‑grade inflammation [10]. The high proportion of asymptomatic or mildly symptomatic cases underscores the importance of routine echocardiographic surveillance in post‑COVID populations, particularly given that subclinical myocardial injury has been documented in a substantial proportion of survivors.

Vaccine‑associated myopericarditis

Our finding that vaccination is associated with increased echocardiographic pericardial abnormality risk (OR 2.08) should be interpreted within the broader context of vaccine safety and efficacy. Multiple population‑based and pharmacovigilance studies have reported a small but significant increase in the risk of myopericarditis following mRNA COVID‑19 vaccination, particularly among males under 40 years and after the second dose [18]. Importantly, the absolute risk remains low, and vaccine‑associated cases are generally mild and self‑limiting, with favorable short‑ and mid‑term outcomes compared with infection‑induced myocarditis [19]. The dose‑response analysis suggests that one or two vaccine doses were associated with significantly higher odds of echocardiographic pericardial abnormalities (OR 2.09 and 2.15, respectively), whereas three or more doses did not show a statistically significant association (OR 1.85, P = 0.118). This pattern may reflect several factors, including limited power in the ≥3‑dose group, longer follow‑up after booster doses, and possible immunomodulatory effects with repeated antigen exposure. The mechanism underlying vaccine‑associated myopericarditis is still being investigated, but current evidence points to an overactivation of innate immunity and interferon‑γ signaling, along with NK‑cell activation, rather than direct viral cytotoxicity [20,21]. Genetic susceptibility and differences in vaccine formulation may further modulate individual risk [21].

Interaction between infection and vaccination

One of the most clinically relevant findings of this study is the significant interaction between COVID‑19 infection and vaccination status in determining echocardiographic pericardial abnormality risk. Among individuals without prior infection, vaccination was associated with increased pericardial involvement odds (OR 2.08, P = 0.003), whereas among those with prior infection, vaccination status did not significantly alter risk (OR 1.01, P = 0.98). Conversely, among unvaccinated individuals, prior COVID‑19 infection increased pericardial involvement odds (OR 1.99, P = 0.012), but this effect was not observed in vaccinated individuals (OR 0.97, P = 0.91). This pattern suggests that both infection and vaccination increase pericardial involvement risk through overlapping inflammatory pathways, such that prior exposure to one stimulus may attenuate the additional risk from the other. The significant interaction for two doses (OR 0.33, P = 0.032) in the dose‑specific model further supports this hypothesis, implying that vaccination‑related inflammatory risk may be lower in those already primed by previous infection. This has potential implications for tailoring vaccination strategies in post‑COVID populations, without undermining the overall benefit of vaccination in preventing severe infection and its complications.

Gender, age, and metabolic factors

Female sex was associated with higher echocardiographic pericardial abnormality prevalence than male sex in this cohort (405/749, 54.1% vs 274/682, 40.2%, P < 0.001), in contrast to reports of male predominance in acute myopericarditis in younger populations [18]. Differences in disease phenotype (isolated pericarditis vs. myopericarditis), age distribution, and referral patterns likely contribute to this discrepancy and highlight the need for sex‑specific analyses in post‑COVID and post‑vaccination cardiovascular syndromes. Age (OR 1.038 per year, P < 0.001) and BMI (OR 1.067 per unit, P < 0.001) were independently associated with echocardiographic pericardial abnormality risk, consistent with the pro‑inflammatory and pro‑atherogenic state of aging and obesity [22,23]. These findings suggest that demographic and metabolic factors should be integrated into risk stratification tools for post‑COVID and post‑vaccination cardiovascular surveillance, particularly in older adults and individuals with obesity or metabolic syndrome.

Clinical implications and risk-benefit considerations

The marked increase in echocardiographic pericardial abnormality diagnoses during pandemic years coincided with the COVID-19 pandemic and may reflect not only biological effects of SARS-CoV-2 infection and vaccination, but also referral bias, greater clinical awareness, lower thresholds for echocardiographic interpretation, and changes in healthcare-seeking behavior and pandemic-era clinical practice. Because the study was retrospective and referral-based, residual confounding cannot be excluded, and the observed associations should be interpreted as correlates rather than evidence of causation. The 2025 ESC Guidelines on myocarditis and pericarditis introduce the concept of inflammatory myopericardial syndrome (IMPS) and advocate systematic cardiovascular evaluation in patients with suspected post‑infection or post‑vaccination inflammatory syndromes [5]. Our findings support the use of echocardiography as a first‑line, low‑cost tool for detecting pericardial effusion, thickening, and hemodynamic compromise in high‑risk individuals, including those with post‑acute sequelae of COVID‑19 or cardiopulmonary symptoms after vaccination [5]. For patients with vaccine‑associated myopericarditis, current evidence indicates that the condition is usually mild and self‑limiting, with generally favorable short‑ and mid‑term outcomes compared with infection‑related myocarditis. Nevertheless, cardiac MRI may reveal persistent myocardial injury in a subset of patients, and long‑term follow‑up is warranted to identify those at risk for chronic cardiomyopathy or recurrent pericarditis [19]. Within the broader risk-benefit framework, the absolute risk of vaccine‑associated myopericarditis remains low, whereas COVID‑19 infection is associated with substantial morbidity and mortality, including severe respiratory illness, multi‑organ dysfunction, and long‑term cardiovascular complications. Public health authorities have consistently concluded that the benefits of COVID‑19 vaccination outweigh the risks across all age groups. Risk‑mitigation strategies include patient education about warning symptoms, careful monitoring in high‑risk subgroups, and informed consent, without compromising overall vaccination coverage.

Underdiagnosis and passive surveillance

A key implication of our findings is the substantial underdiagnosis of inflammatory myopericardial syndromes in routine clinical practice. Nearly 70% (463/673, 68.8%) of patients undergoing echocardiography during pandemic years had pericarditis findings, suggesting that many cases are asymptomatic or mildly symptomatic, escaping passive surveillance systems that rely on spontaneous reporting [5]. Passive pharmacovigilance systems capture only a fraction of adverse events, especially those with nonspecific or mild symptoms. International studies report lower prevalence of clinically defined myocarditis and pericarditis, depending on follow‑up timing and disease severity. For example, Puntmann et al. [6] reported myocardial injury or inflammation in ≈78% of patients one to two months after COVID‑19 using cardiac MRI, whereas Chamtouri et al. [14] identified myocarditis or myocardial injury in ≈40% of recovered patients at one, two, or four months after infection. However, these studies focused on myocardial involvement and clinically overt disease, often within short follow‑up windows. By contrast, our cohort reflects systematic echocardiographic screening in an outpatient cardiology clinic, where even minimal pericardial effusions and subclinical involvement are recorded as markers of prior inflammatory exposure. We also included chronic and residual pericarditis, extending the window of detection well beyond the acute or early post‑infective phase. A recent meta‑analysis by Cotet et al. [24] reported myocarditis prevalence ≈1.3-4.0% in COVID‑19 cohorts but excluded isolated pericarditis and did not systematically consider subclinical findings or echo‑only diagnoses. In contrast to these predominantly infection‑focused cohorts, our study reflects a real‑world outpatient setting in which both post‑COVID and post‑vaccination cardiovascular sequelae were captured over an extended follow‑up period. The inclusion of minimal pericardial effusions and residual echocardiographic findings-often overlooked in registries based on clinical symptoms or MRI‑defined myocarditis-may partially explain our higher prevalence estimates. The prognostic significance of these minimal echo findings remains uncertain, underscoring the need for long‑term follow‑up to determine whether they truly represent a “severe cardiovascular risk factor” or simply a benign sequel of prior inflammation in the real‑world post‑pandemic landscape.

Limitations

Several limitations warrant consideration. First, the retrospective design precludes causal inference, and residual confounding cannot be excluded despite multivariable adjustment. Second, because the study was conducted in a referral-based outpatient echocardiography cohort, selection bias and referral bias are possible, and the findings may not be generalizable to broader populations. Third, increased clinical awareness of COVID-19-related cardiac complications during the pandemic may have influenced referral patterns, echocardiographic interpretation, and diagnostic thresholds over time, thereby contributing to detection bias and to the marked temporal increase observed in pericardial abnormalities. In addition, COVID-19 infection and vaccination status were obtained from clinical records and registries, with the possibility of exposure misclassification due to asymptomatic or undocumented infections and the lack of detailed information on the exact timing of infection or vaccination relative to echocardiography. This limited our ability to establish precise temporal relationships between exposure and imaging findings, and therefore any causal interpretation should be avoided. As this was a retrospective cohort study, the analysis was limited to the covariates that were consistently available in the clinical record and could be reliably extracted for all patients. Consequently, no further sensitivity analyses based on additional patient-level variables or alternative adjustment strategies were feasible beyond the models reported here. Finally, the outcome was based on echocardiographic findings rather than systematic clinical, laboratory, or multimodality imaging confirmation of pericardial inflammation. Although this inclusive definition increased sensitivity for detecting minimal abnormalities, it may also have reduced clinical specificity and inflated the apparent burden of inflammatory disease. The prognostic significance of minimal or residual echocardiographic abnormalities remains uncertain, and further prospective studies using standardized diagnostic criteria are needed to clarify their clinical relevance.

Strengths

Despite these limitations, the study has important strengths. It is based on real‑world data from a large, consecutive outpatient echocardiography cohort, spanning both pre‑pandemic and pandemic periods. The long follow‑up window and systematic echo screening allowed detection of both post‑COVID and post‑vaccination inflammatory myopericardial syndromes, including asymptomatic and minimally symptomatic cases not captured in event‑driven registries.

Future directions

Future research should focus on prospective longitudinal cohorts with standardized diagnostic criteria for pericardial disease, integrating symptoms, ECG findings, inflammatory biomarkers, and cardiac magnetic resonance imaging to improve diagnostic specificity and reproducibility. Such studies are needed to clarify whether minimal echocardiographic pericardial abnormalities represent transient or clinically meaningful inflammatory sequelae, and to define their prognostic significance over time. In addition, future investigations should evaluate the temporal relationship between SARS-CoV-2 infection, vaccination, and echocardiographic findings, ideally incorporating precise exposure timing, vaccine formulation, infection severity, and disease course. Sex-, age-, and BMI-stratified analyses may also help elucidate the biological mechanisms underlying the observed associations and identify subgroups at higher risk.

## Conclusions

In this five-year retrospective cohort, both COVID-19 infection and SARS-CoV-2 vaccination were associated with increased odds of echocardiographic pericardial abnormalities. These findings should be interpreted as associative rather than causal, particularly because the outcome included minimal residual echocardiographic abnormalities of uncertain clinical relevance and the study was subject to referral-based sampling. Prospective validation with standardized diagnostic criteria and comprehensive confounder adjustment is required.
